# Anti-CDC25B autoantibody predicts poor prognosis in patients with advanced esophageal squamous cell carcinoma

**DOI:** 10.1186/1479-5876-8-81

**Published:** 2010-09-03

**Authors:** Jun Dong, Bo-hang Zeng, Li-hua Xu, Jun-ye Wang, Man-Zhi Li, Mu-sheng Zeng, Wan-li Liu

**Affiliations:** 1 The Second Affiliated Hospital of Guangzhou Medical University, Gyangzhou, China; 2 Department of Experimental Research, Sun Yat-sen University Cancer Center, Guangzhou, China; 3 State Key Laboratory of Oncology in South China and Department of Thoracic, Sun Yat-sen University Cancer Center, Guangzhou, China; 4 Department of Thoracic, Sun Yat-sen University Cancer Center, Guangzhou, China; 5 Department of Clinical Laboratory Medicine, Sun Yat-sen University Cancer Center, Guangzhou, China

## Abstract

**Background:**

The oncogene CDC25B phosphatase plays an important role in cancer cell growth. We have recently reported that patients with esophageal squamous cell carcinoma (ESCC) have significantly higher serum levels of CDC25B autoantibodies (CDC25B-Abs) than both healthy individuals and patients with other types of cancer; however, the potential diagnostic or prognostic significance of CDC25B-Abs is not clear. The aim of this study is to evaluate the clinical significance of serum CDC25B-Abs in patients with ESCC.

**Methods:**

CDC25B autoantibodies were measured in sera from both 134 patients with primary ESCC and 134 healthy controls using a reverse capture enzyme-linked immunosorbent assay (ELISA) in which anti-CDC25B antibodies bound CDC25B antigen purified from Eca-109 ESCC tumor cells. The clinicopathologic significance of CDC25B serum autoantibodies was compared to that of the tumor markers carcinoembryonic antigen (CEA), squamous cell carcinoma antigen (SCC-Ag) and cytokeratin 19 fragment antigen 21-1(CYFRA21-1).

**Results:**

Higher levels of CDC25B autoantibodies were present in sera from patients with ESCC (A_450 _= 0.917, SD = 0.473) than in sera from healthy control subjects (A_450 _= 0.378, SD = 0.262, *P *< 0.001). The area under the receiver operating characteristic (ROC) curve for CDC25B-Abs was 0.870 (95% CI: 0.835-0.920). The sensitivity and specificity of CDC25B-Abs for detection of ESCC were 56.7% and 91.0%, respectively, when CDC25-Abs-positive samples were defined as those with an A_450 _greater than the cut-off value of 0.725. Relatively few patients tested positive for the tumor markers CEA, SCC-Ag and CYFRA21-1 (13.4%, 17.2%, and 32.1%, respectively). A significantly higher number of patients with ESCC tested positive for a combination of CEA, SCC, CYFRA21-1 and CDC25B-Abs (64.2%) than for a combination of CEA, SCC-Ag and CYFRA21-1 (41.0%, *P *< 0.001). The concentration of CDC25B autoantibodies in serum was significantly correlated with tumor stage (*P *< 0.001). Although examination of the total patient pool showed no obvious relationship between CDC25B autoantibodies and overall survival, in the subgroup of patients with stage III-IV tumors, the cumulative five-year survival rate of CDC25B-seropositive patients was 6.7%, while that of CDC25B-seronegative patients was 43.4% (*P *= 0.001, log-rank). In the N1 subgroup, the cumulative five-year survival rate of CDC25B-seropositive patients was 13.6%, while that of CDC25B-seronegative patients was 54.5% (*P *= 0.040, log-rank).

**Conclusions:**

Detection of serum CDC25B-Abs is superior to detection of the tumor markers CEA, SCC-Ag and CYFRA21-1 for diagnosis of ESCC, and CDC25B-Abs are a potential prognostic serological marker for advanced ESCC.

## Background

Esophageal squamous cell carcinoma (ESCC), the major histopathological form of esophageal cancer, is one of the most lethal malignancies of the digestive tract and is the fourth most frequent cause of cancer deaths in China [1]. Despite the improvements in surgical techniques and adjuvant chemoradiation for patients with ESCC, the five-year survival rate of patients with advanced ESCC is still poor [2]. This poor survival rate is largely due to the lack of serological markers for early diagnosis and prediction of disease progression; patients are frequently diagnosed with ESCC when they have already reached an advanced stage of disease [3]. There is thus a growing need to identify useful biological markers for early, non-invasive diagnosis of ESCC and for monitoring tumor progression [4].

In addition to the traditional tumor markers CEA, SCCA and CYFRA21-1, autoantibodies against tumor-associated antigens were recently reported in sera from patients with ESCC. Similar to the traditional tumor markers, these autoantibodies were shown to be useful molecular markers for ESCC. Some patients with ESCC mount an immunological reaction against several tumor-associated antigens, including p53 [5-7], myomegalin [8] and TRIM21 [9]. Recently, a proteomics-based approach identified several autoantibodies in sera of patients with ESCC, such as anti-heat shock protein 70 [10] and anti-peroxiredoxin VI [11]. The presence of these autoantibodies in sera has been reported as a useful marker for early diagnosis or for prediction of disease progression in patients with ESCC.

Most recently, we identified CDC25B autoantibodies in sera from patients with ESCC using a proteomics-based technique[12]. Three CDC25B phosphatases exist in higher eukaryotes, CDC25A, CDC25B and CDC25C[13]. CDC25B has been shown to play an important role in tumorigenesis [14]. First, CDC25B can transform fibroblast cells lacking functional retinoblastoma protein or harboring mutated Ras protein[15]. Second, CDC25B activates the mitotic kinase CDK1/cyclin B complex in the cytoplasm to stimulate cell cycle progression [16]. Furthermore, overexpression of CDC25B has been observed in a variety of human cancers, including colon cancer[17], medullary thyroid carcinoma [18], breast cancer [19], non-Hodgkin's lymphomas[20], non-small cell lung cancer [21] and ESCC[22-25]. We previously reported that aberrant expression of CDC25B in ESCC tumor cells can induce CDC25B autoantibodies in sera of ESCC patients, and antibodies against CDC25B were detected in sera of 36.3% of patients with ESCC, but not in sera of healthy controls, by reverse capture ELISA [12]. Our findings suggest that CDC25B autoantibodies are a novel serum marker for ESCC.

Although higher levels of anti-CDC25B antibodies were found in the sera of patients with ESCC than in the sera of healthy controls, the relationship between tumor burden, tumor staging and antibody levels remains unknown. In addition, the potential utility of anti-CDC25B antibodies for diagnosis of ESCC has not been clearly addressed. In this study, we established a reverse capture ELISA to detect anti-CDC25B antibodies in sera from patients with ESCC and evaluated the clinical values of CDC25B autoantibodies for diagnosis of ESCC and prediction of tumor progression.

## Methods

### Patients and sera

Sera were collected from 134 patients with primary ESCC at the time of diagnosis before tumor resection at the Cancer Center of Sun Yat-sen University between January 2003 and December 2004. Ninety-three patients were male and 41 patients were female. The patients ranged in age from 38 to 81 years (mean, 58.5 years), and none of them had received radiation therapy or chemotherapy before surgery. Sera from 134 healthy volunteers (91 males and 43 females with ages ranging from 40 to 70 years (mean, 61 years)) were collected and used as controls. Prior to the use of these sera, informed consent was obtained from patients and experiments were approved by the Institute Research Ethics Committee. After collection, sera were immediately aliquoted and stored at -80°C until use.

### Cell lines

The ESCC cell lines Eca-109, TE-1, and Kyse140 (Cell Bank of Type Culture Collection of Chinese Academy of Sciences, Shanghai, China) were grown in RPMI 1640 (Invitrogen, Carlsbad, CA) supplemented with 10% fetal bovine serum, 100 μg/L streptomycin, and 100 μg/L penicillin in a humidified incubator containing 5% CO_2 _at 37°C. The immortalized esophageal cell line NE-3 was obtained from Dr. Jin (the University of Hong Kong, P. R. China)[26] and cultured in Keratinocyte-SFM (Invitrogen, Carlsbad, CA).

### Western blot analysis

Western blots were performed as described previously [27]. The membranes were stained with a 1:1000 dilution of an anti-CDC25B antibody (Cell Signaling Technology, Danvers, MA) or with a 1:2000 dilution of a mouse monoclonal anti-α-tubulin antibody (Santa Cruz Biotechnology, Santa Cruz, CA). A non-tumorous tissue protein was obtained from a patient with ESCC who underwent surgical esophageal tissue resection at the Cancer Center of Sun Yat-sen University (Guangzhou, P. R. China) during 2009 and used as a negative control.

### Preparation of Antigen Protein

Antigen protein was extracted from the ESCC cell lines and prepared as reported previously [28]. Briefly, after washing the cells three times with phosphate-buffered saline (PBS), the cells were collected and incubated at a concentration of 10^7 ^cells/ml in a lysis buffer composed of Tris base (10 mmol/L), NaCl (150 mmol/L), Triton-X (0.1%) and a proteinase inhibitor cocktail, placed on ice, vortexed every 10 min for 1 h, and centrifuged at 10,000 × g for 20 min at 4°C. The supernatant was then collected as an antigen protein sample and stored at -80°C until use. The final protein concentration was determined using a BCA protein assay kit (Thermo Fisher Scientific, Fremont, CA).

### Reverse capture ELISA for Detection of CDC25B Autoantibodies

A 96-well plate (Costar) was coated overnight with purified anti-CDC25B monoclonal antibody (100 ng/well in 50 mM bicarbonate buffer (pH 9.0), Cell Signaling Technology, Danvers, MA) at 4°C. Wells were then blocked for 2 h at 37°C with 3% bovine serum albumin (BSA) in PBS. The antigen protein sample was diluted in PBS (pH 7.0) to final concentrations of 20 mg/ml, 10 mg/ml and 5 mg/ml, added to blocked wells (100 μl/well) and incubated overnight at 4°C. Wells were then washed three times with PBST (0.1% (v/v) Tween 20 in PBS), and the 100 μl serum samples (1:200 dilution with PBST) were incubated in the wells for 2 h at 37°C. Rabbit anti-human CDC25B polyclonal antibody (1:10,000 dilution in PBST, Abcam) was used as a positive control, and 3% BSA served as a negative control. After washing the wells four times with PBST, each well was incubated with a 1:10,000 dilution of 100 μl goat anti-human or anti-rabbit IgG-HRP conjugate (Santa Cruz Biotechnology, Santa Cruz, CA) for 1 h at 37°C. The wells were then washed with PBST and incubated with TMB developing reagent for 5 min in the dark. The reactions were stopped with 0.5 mol/L H_2_SO_4 _and the absorbance of each well was measured at 450 nm using a Multiskan Spectrum plate reader (Thermo LabSystems). Sera from ESCC patients and healthy volunteers were tested simultaneously, and each sample was assayed twice in duplicate wells.

### CEA, SCC and CYFRA21-1 Assay

Serum CEA and CYFRA21-1 were assessed by an electrochemiluminescence immunoassay using E170 analyzer (Roche Diagnostics Gmbh, Roche, USA). Serum SCC-Ag was measured by a microparticle enzyme immunoassay (ABBOTT Diagnostics, Abbott, USA).

### Statistical Analysis

All statistical analyses were performed using the SPSS 16.0 software package. The cut-off value for seropositivity of CDC25B-Abs was identified by the ROC curve. Pearson's chi-square test or Fisher's exact test was employed to assess the association between CDC25B seropositivity and clinicopathologic characteristics. The statistical difference in CDC25B-Abs levels between patients with tumors and healthy control subjects was evaluated using the Mann-Whitney U test. Survival curves were estimated by Kaplan-Meier plots and log-rank tests. Cox proportional hazard regression analysis was used to estimate the hazard ratios of independent factors for survival. P < 0.05 in all case was considered statistically significant.

## Results

### Anti-CDC25B autoantibodies in sera of patients with ESCC

**Figure 1:**
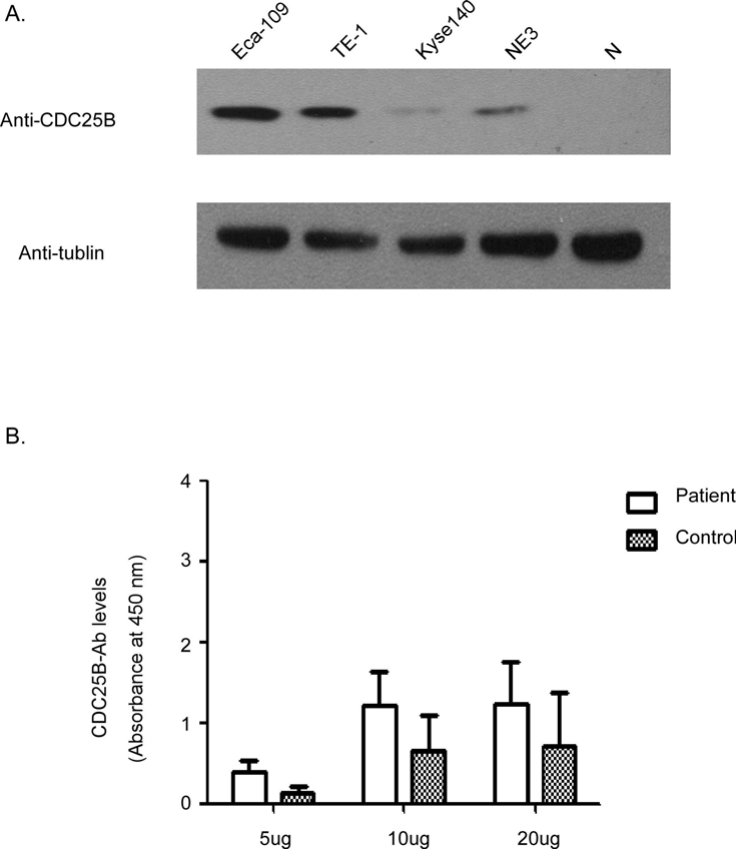
**Expression of CDC25B in different ESCC cell lines and optimization of antigen concentration for ELISA assays**. A. Expression of CDC25B in ESCC cell lines was examined by Western blot analysis with an anti-CDC25B antibody (N: normal esophageal tissue). B. Effect of different amounts of Eca-109 total protein on absorbance at 450 nm in CDC25B-Abs reverse capture ELISA. Twenty samples from patients with ESCC and twenty samples from healthy controls were tested in reverse capture ELISAs using different amounts of Eca-109 total protein as antigen. The results shown are the mean values of three experiments.

**Figure 2:**
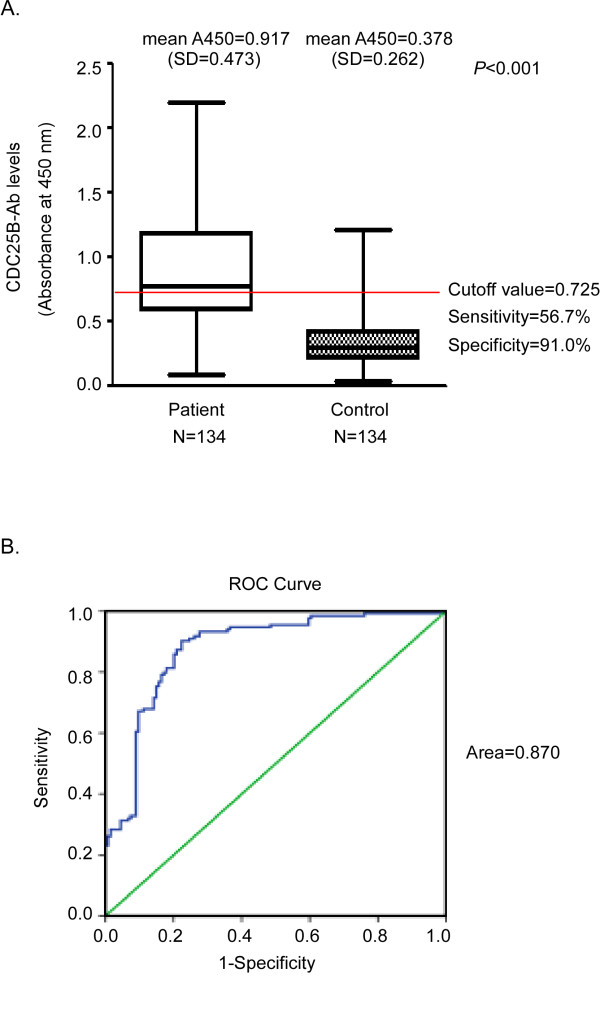
**CDC25B autoantibodies in sera from patients with ESCC and healthy controls and ROC Curve analysis**. A. CDC25B-Abs were detected by reverse capture ELISA in sera from patients with ESCC (Patient) and healthy controls (Control). The *horizontal line *indicates the cut-off value used to define positive samples. The results shown are the mean values of two independent experiments. B. ROC curve of CDC25B-Abs in sera from patients with ESCC. The area under the ROC curve is 0.870. The cut-off value is determined according to the ROC curve.

One hundred thirty-four patients with ESCC were enrolled in the study (Table 1). The presence of CDC25B autoantibodies in sera of ESCC patients was assessed by reverse capture ELISA. The extract of Eca-109 cells, which presented the highest CDC25B protein level among the ESCC tumor cell lines tested (Eca-109, Kyse140, TE-1 and the immortalized cell line NE-3) (Figure 1A), was used as the source of CDC25B antigen for reverse capture ELISAs. To determine the optimal amount of Eca-109 cell extract for use in these assays, 20 sera samples from ESCC patients and 20 sera samples from healthy controls were evaluated by reverse capture ELISA. As shown in Figure 1B, 10 μg/well of total Eca-109 cell protein was determined to be the optimal protein concentration. The within-run coefficient of variation (CV) for a patient sample (OD 1.35) and a healthy control sample (OD 0.23) were 10.3% and 9.1%, respectively, as determined by repeating the assay 20 times. Under these conditions, the average absorbance was 0.378 (SD = 0.262) in sera from 134 healthy control subjects and 0.917 (SD = 0.473) in sera from 134 patients with primary ESCC (Figure 2A). The circulating levels of CDC25B-Abs in patients with ESCC were significantly higher than those of healthy control subjects (*P *< 0.001).

**Table 1:** Association between the clinical pathologic features of ESCC and the presence of CDC25B-Abs

			CDC25B-Abs		
				
Characteristics	Total (n = 134)	OD(SD)	Positive cases n (%)	Negative cases n (%)	*P*
Gender					
Male	93	0.913(0.496)	48 (51.6)	45 (48.4)	0.053
Female	41	0.921(0.431)	28 (68.3)	13 (31.7)	
Age (y)					
<60	73	0.871(0.491)	44 (60.3)	29 (47.5)	0.231
≥60	61	0.968(0.458)	32(52.5)	29 (39.7)	
Stage					
I-II	80	0.892(0.478)	37 (46.3)	43 (53.7)	0.002
III-IV	54	0.958(0.481)	39 (72.2)	15 (27.8)	
pT classification					
T1-T2	48	0.904(0.482)	28 (58.3)	20 (41.7)	0.461
T3-T4	86	0.919(0.477)	48 (55.8)	38 (44.2)	
pN classification					
YES	53	0.980(0.511)	29 (54.7)	24 (45.3)	0.420
NO	81	0.873(0.451)	47 (58.0)	34 (42.0)	
Metastasis					
YES	8	0.917(0.436)	3 (37.5)	5 (62.5)	0.222
NO	126	0.932(0.484)	73 (57.9)	53 (42.1)	

OD: optical density

### Sensitivity and specificity of serum CDC25B-Abs, CEA, SCC and CYFRA21-1 in detection of ESCC

The ROC curve was plotted to identify a cut-off value that would distinguish ESCC from nonmalignant esophageal diseases. According to the ROC curve, the optimal cut-off value was 0.725, providing a sensitivity of 56.7% and a specificity of 91.0%. The area under the ROC curve for CDC25B-Abs was 0.870 (95% CI: 0.835-0.920; Figure 2B). CDC25B-Abs were found in sera from 76 of 134 (56.7%) patients with ESCC, but in sera from only 11 of 134 (8.2%) healthy controls. Serum CDC25B-Abs were detected in a higher proportion of patients with ESCC than healthy control subjects (*P *< 0.001, Figure 2A); however, sera from only 17.2% of ESCC patients contained SCC-Ag at levels above the cut-off value of 1.5 ng/ml, 13.4% of ESCC patients contained CEA at levels above the cut-off value of 5.0 ng/ml and 32.1% of ESCC patients with the sera CYFRA21-1 levels above the cut-off value of 3.5 ng/ml. (Table 2). These data indicate that the percentage of CDC25B-Abs seropositivity in patients with ESCC is dramatically higher the percentages of seropositivity of the previously described tumor markers SCC-Ag, CEA and CYFRA21-1 in these patients. In addition, sera from 41.0% of patients with ESCC were positive for CEA, SCC-Ag or CYFRA21-1, while sera from 64.2% of patients with ESCC were positive for CEA, SCC-Ag, CYFRA21-1 or CDC25B-Abs (Table 2). The sensitivity of these four markers used in combination was slightly higher than that of the CDC25B-Abs marker alone but significantly higher than that of CEA, SCC-Ag and CYFRA21-1 used in combination (*P *< 0.001).

**Table 2:** The sensitivity of CDC25B-Abs, CEA, CYFRA21-1 and SCC-Ag in detection ESCC.

Tumor Markers	Total n	Positive	Negative	
			
		n (%)	n (%)	*P*
CDC25B-Abs	134	76 (56.7)	58 (43.3)	
SCC-Ag	134	23 (17.2)	111 (82.8)	
CEA	134	18 (13.4)	116 (86.6)	
CYFRA 21-1	134	43 (32.1)	91 (67.9)	
CEA, SCC-Ag	134	55 (41.0)	79 (59.0)	
or CYFRA21-1				
CEA, SCC-Ag,	134	86 (64.2)	48 (35.8)	<0.001*
CYFRA21-1 or CDC25B-Abs				

Abs: antibodies; CEA: carcinoembryonic antigen; SCC-Ag: squamous cell carcinoma antigen; CYFRA21-1: cytokeratin 19 fragment antigen 21-1.

*compared with either CEA, SCC-Ag or CYFRA21-1. Cut-off values: 5.0 ng/ml for CEA; 1.5 ng/ml for SCC-Ag; 3.5 ng/ml for CYFRA21-1

### Association between CDC25B-Abs and Clinicopathological Characteristics

The data presented in Table 1 show the relationship between CDC25B-Abs and clinicopathological variables in ESCC. CDC25B-Abs were not obviously correlated with T classification, N classification or metastasis; however, there was a significant association between the presence of CDC25B-Abs and ESCC clinical stage (*P *= 0.002). The percentage of CDC25B-Abs seropositivity was higher in patients with advanced disease than in patients with early disease.

### Association of CDC25B-Abs with Survival

**Figure 3:**
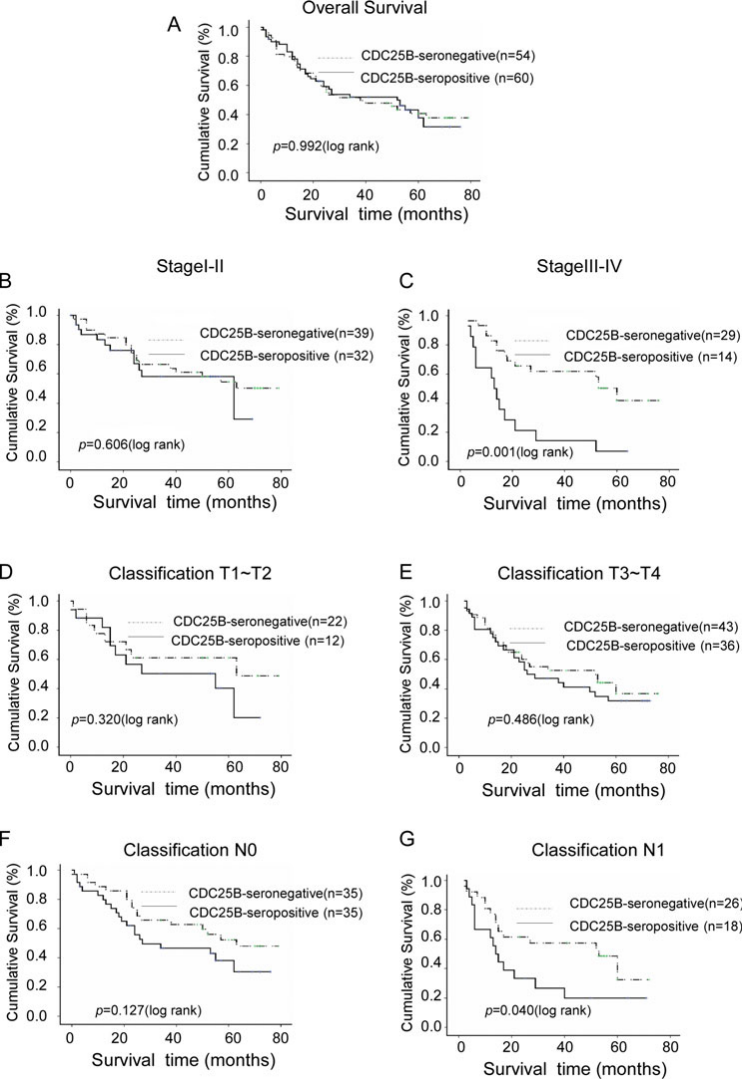
**Kaplan-Meier curves with univariate analyses (log-rank) for patients with positive CDC25B expression versus negative CDC25B expression**. The five-year survival rates of seropositive (bold line) and seronegative (dotted line) ESCC patients are not significantly different (A, *P *= 0.992, log-rank). The survival rates of CDC25B-seropositive and CDC25B-seronegative patients were compared in subgroups with stage I-II (B) and stage III-IV ESCC (C). The same comparison was carried out in patients classified into the T1-T2 (D), T3-T4 (E), N0 (F) and N1 (G)groups. *P *values were calculated using the log-rank test.

The overall survival of ESCC patients was plotted using the Kaplan-Meier method, and a log-rank test was employed to evaluate the prognostic significance of CDC25B-Abs. There was no statistical difference between the survival rate of the CDC25B-seronegative patients and that of the CDC25B-seropositive patients (*P *= 0.992) (Figure 3A). We then analyzed the potential prognostic value of CDC25B-Abs in different subgroups of patients stratified according to the clinical stage of the patient's tumor, T classification and N classification. As shown in Figure 3, for the subgroup with clinical stage III-IV tumors, the cumulative five-year survival rate was 43.4% in the CDC25B-seronegative patients and 6.7% in the CDC25B-seropositive patients (*P *= 0.001, log-rank). In a similar analysis of the N1 subgroup, the cumulative five-year survival rate was 54.5% in the CDC25B-seronegative patients and 13.6% in the CDC25B-seropositive patients (Figure 3G) (*P *= 0.040, log-rank). In addition, multivariate survival analysis was used to determine whether circulating CDC25B-Abs were an independent prognostic factor. Our results showed that the level of circulating CDC25B-Abs had a significant relationship with the prognosis of patients with advanced ESCC (*P *= 0.001) (Table 3); however, the difference between CDC25B-seropositive patients and CDC25B-seronegative patients was not statistically significant in patients classified into the stage I-II (*P *= 0.606, log-rank; Figure 3B), T1-T2 (*P *= 0.320, log-rank; Figure 3D), T3-T4 (*P *= 0.486, log-rank; Figure 3E) and N0 (*P *= 0.127, log-rank; Figure 3F) subgroups.

**Table 3:** Univariate and multivariate analysis of different prognostic parameters in ESCC patients in the N1 subgroup by Cox regression analysis.

	Univariate analysis		Multivariate analysis			
	
	No. patients	*P*	Regression coefficient(SE)	*P*	Relative risk	95% confidence interval
pT classification						
T1-T2	7	0.015	0.405(0.196)	0.040	1.499	1.020-2.202
T3-T4	37					
CDC25B-Abs						
Seronegative	18	0.001	0.633(0.196)	0.001	1.882	1.283-2.762
Seropositive	26					

## Discussion

The identification of tumor antigens that elicit an immune response is important for clinical applications; tumor antigens may used for early diagnosis, prognosis, and immunotherapy against the disease[29]. In this study, we show that CDC25B-Abs in sera from ESCC patients were more sensitive than CEA, SCC-Ag and CYFRA21-1 for diagnosis of ESCC. Moreover, serum levels of CDC25B-Abs were correlated with the clinicopathologic characteristics present in patients with advanced ESCC.

CEA, SCC-Ag and CYFRA21-1 have been used as tumor markers for diagnosis of ESCC [30]. However, reliance on the three tumor markers for the detection of ESCC has not been satisfactory, especially because of the poor sensitivity of these tumor markers for ESCC[31]. In line with previous studies, our current study showed that the sensitivity of CEA, SCC-Ag or CYFRA21-1 for detection of ESCC was less than 35%[32-34]. To circumvent the problem of low sensitivity, we and others have begun to evaluate the use of autoantibodies against tumor antigens to detect ESCC. Ralhan has shown that anti-p53 antibodies were found in 60% sera from patients with ESCC[5], and Shimada has reported that anti-p53 antibodies were found in 40% sera from patients with ESCC and surveillance of serum p53-Abs was superior to CEA, SCC-Ag and CYFRA21-1 [6]. Autoantibody against Prx VI was found in sera from 50% of patients with ESCC[11]. Serum anti-myomegalin antibodies were present in 47% of patients with ESCC [8]. Our previous study showed that 36.3% of ESCC patients with autoantibody responses to CDC25B [12]. These results suggest that autoantibodies increase the sensitivity of detection of ESCC and might be useful tumor markers for ESCC diagnosis.

In the current study, CDC25B autoantibodies were detected in sera of ESCC patients by reverse capture ELISA. This technology is based on capturing specific antigens from tumor cell lysates with antibodies, allowing the antigens to be immobilized in their native configuration [35-37]. Due to optimization of the reverse capture ELISA in current study, the sensitivity of this assay is higher than in our previous report (36.3%), but its specificity is lower than that reported in our previous study (100%) [12]. The rate of CDC25B-Abs seropositivity in patients with ESCC was significantly higher than the seropositivity rates of tumor markers SCC-Ag, CEA and CYFRA21-1. Moreover, the combination of CDC25B-Abs and conventional tumor markers, CEA, SCC-Ag, and CYFRA21-1 significantly increased the sensitivity of detection of ESCC. Our data suggest that CDC25B-Abs could be a potential biomarker for ESCC diagnosis.

In addition, our results demonstrate that CDC25B autoantibodies were more prevalent in sera from patients with advanced ESCC than in sera from patients with early stage disease (*P *< 0.001) and that in the patients with clinical stage III-IV and N1 subgroup, CDC25B-Abs seronegative patients survived longer than CDC25B-Abs seropositive patients. This observation may be explained by the higher incidence of CDC25B overexpression in advanced ESCC than in early stage tumors[22,25]. CDC25B protein expression increased as tumors progressed; none of the healthy control subjects expressed CDC25B, while one-fourth of the dysplasia subjects and one-half of the patients with invasive cancer expressed CDC25B[25]. Moreover, overexpression of CDC25B was also more frequently found in patients with deep tumor invasion and lymph node metastasis than in patients with early stage disease [22,38]. Overexpression of CDC25B in advanced ESCC may thus lead to high production of CDC25B-Abs in patients with advanced tumors. These results suggest that detection of serum CDC25B-Abs is a useful non-invasive marker for identifying advanced ESCC patients with poor prognosis.

In summary, the levels of CDC25B-Abs in sera from ESCC patients were significantly higher than those in sera from healthy subjects. Detection of CDC25B-Abs in combination with CEA, SCC-Ag, CYFRA21-1 results in significantly increased sensitivity of detection, with 64.2% of ESCC patients testing positive for at least one of these markers. Moreover, our study has demonstrated the prognostic significance of serum CDC25B-Abs in ESCC and the clinical implications of CDC25B-Abs seropositivity on lymph node metastasis and advanced stage ESCC. High levels of CDC25B autoantibodies in sera were significantly associated with poor survival in advanced ESCC. CDC25B autoantibodies are thus a useful prognostic predictor for advanced ESCC.

## Conclusions

Our findings indicate that the levels of CDC25B-Abs in sera from patients with ESCC are significantly higher than those of other tumor markers. Moreover, high levels of CDC25B-Abs were associated with poor survival in advanced ESCC. Multivariate survival analysis showed that CDC25B-Abs are a potential prognostic serological marker for advanced ESCC. CDC25B-Abs therefore provide a valuable serological marker in the prognostic evaluation of advanced ESCC.

## Abbreviations

ESCC: esophageal squamous cell carcinoma; ELISA: enzyme-linked immunosorbent assay; CEA: carcinoembryonic antigen; SCC-Ag: squamous cell carcinoma antigen; ROC: receiver operating characteristic; PBS: phosphate-buffered; OD: optical density; CDK: cyclin-dependent kinase; CYFRA21-1: cytokeratin 19 fragment antigen 21-1

## Competing interests

The authors declare that they have no competing interests.

## Authors' contributions

MSZ is responsible for the study design. JD and BHZ performed the experiments and drafted the manuscript. LHX participated in the data analysis and Western blots. All authors read and approved the final manuscript.
